# The London Hospital

**Published:** 1908-02-08

**Authors:** 


### HOSPITAL ADMINISTRATION.

### CONSTRUCTION AND ECONOMICS.

### THE LONDON HOSPITAL.

#### II.—ITS FINANCIAL NEEDS AND HOW TO MEET THEM.

Last week we dealt with the development and present condition of the London Hospital, and also published some historical facts which have excited general interest. To-day we propose to consider the financial position of this great institution. Twenty years ago the financial condition of all the voluntary hospitals of London was unsound, and there were many people who, whilst believing in the principle of voluntary support, feared that the added cost of modern methods of treatment, and the demand for better and more costly buildings, would render it necessary that these hospitals should have in some form pecuniary aid from the State or the municipality. Owing, however, to the active personal interest taken in the voluntary hospitals by Queen Victoria, and in recent years by King Edward VII., the Prince of Wales, and every members of the Royal Family, together with the organised and continuous efforts which have been made to widen the area of givers and to bring in much larger contributions from voluntary sources, the financial position of the great hospitals in this country was never stronger than it is to-day.

In the first chapter of Burdett's *Hospitals and Charities* for 1908, which will be issued in a few days, there are exhaustive tables and audited figures, which prove the precise accuracy of the statement we have just made. Thus in 1896 the ordinary income of 716 hospitals in the United Kingdom amounted to one million nine hundred and thirty thousand pounds in round figures. In 1905 the ordinary income of 794 hospitals had increased to two millions four hundred and fifty-six thousand pounds; special donations increased from ninety-six thousand pounds to five hundred and sixty thousand pounds, and legacies showed an increase of upwards of three hundred and seventy-six thousand pounds. Dealing with the Metropolitan hospitals alone, ninety-nine such institutions had an ordinary income in 1896 of six hundred and forty-six thousand pounds. The ordinary income of one hundred and five such hospitals in 1905 amounted to seven hundred and ninety-four thousand pounds. The legacies increased by two hundred and forty-eight thousand pounds, and the special donations by upwards of two hundred thousand pounds. It further appears that, whereas the expenditure of these Metropolitan hospitals in 1896 was sixty-one thousand pounds more than the revenue from all sources, in 1905 there was a surplus revenue of nearly ninety-one thousand pounds.

We are led to give these figures in the present article because we want to make it perfectly clear that the volume of charity from voluntary sources, if properly distributed, is adequate to meet the requirements of all the hospitals in the Metropolis at the present time. Of course, each year some hospitals do relatively badly, whilst others do relatively well; but on the whole, the actual money contributed by the public is enough to defray the total expenditure of all the hospitals each year. In these circumstances it is evident that the largest of all the Metropolitan hospitals should certainly be provided with an adequate revenue to meet fully every possible expenditure.

Let us next consider what the actual financial position of the London Hospital is to-day, as indicated by its estimated income and estimated expenditure of a given year. First of all it may be well to state that the system pursued has been so businesslike that to-day the loans on mortgage—that is, the actual balance of liability due to the large expenditure rendered necessary by the rebuilding of the hospital—only amount to the relatively small sum of £18,600. We take it that the statement of this fact should commend itself to some man of wealth, for then he may be moved to send Mr. Holland a cheque for the whole amount, and so close this account once and for all.

Turning next to income and expenditure, we find that the estimated expenditure each year is £100,000 in round figures. To meet this the estimated income from all sources is £74,500, made up as follows:—

| | |
|---|---|
| Annual Subscriptions ... ... ... ... ... | £15,000 |
| Donations for Maintenance (including Peoples' Subscription Fund and Forget-me-not Bond) ... | 10,000 |
| King's Fund ... ... ... ... ... ... ... | 8,000 |
| Sunday Fund ... ... ... ... ... ... ... | 5,500 |
| Saturday Fund ... ... ... ... ... ... ... | 1,000 |
| Income from Invested Property and Trust Funds | 16,000 |
| Private Nursing and Training School Fees ... ... | 7,000 |
| Legacies, an average, say, of ... ... ... ... ... | 12,000 |

The hospital has, therefore, to obtain at least £25,500 each year from special sources, or £127,500 in the whole. It will be seen, however, that to prove effective the present appeal for a five years' Sustentation Fund should result in the raising of practically £150,000. We have no doubt that this money will be forthcoming, but we should like to see an effort made so to readjust the scheme of finance as to make the present appeal result in an increased income to the London Hospital of £30,000 a year. If this additional income can be raised the institution will be permanently financed, and its work can be efficiently carried on without the necessity for any further quinquennial appeals such as that which is being made at the present time.

We were looking into the figures of the London Hospital recently, and we find that about one-third of the money obtained from previous quinquennial appeals may be regarded as being capable of being retained as permanent revenue. Assuming that this estimate is correct, it will be necessary in some way to devise means which will bring to the London Hospital from other sources a permanent additional revenue of £20,000. To obtain this money at the least cost with the greatest facility experience shows that various fields should be cultivated, so that the work of raising the money may be minimised as much as possible, and the widest possible volume of sympathy evoked in favour of the hospital. There are at the present time some five hundred students at the London Hospital, and its medical school has a good reputation, and has sent out into the profession thousands of medical men. We believe that if a list of the men who received their medical education at the London Hospital was to be prepared, and an active committee selected from them to issue an appeal to their fellows, pointing out that a determined attempt is being made to finance the London Hospital, and that if they collectively could raise £5,000 a year in annual contributions the hospital's finances would be placed in a sound position, this sum would be readily forthcoming. That would leave £15,000 to be obtained from other sources. We would suggest that the committee, which is a large and influential body of representative men, might agree to raise amongst themselves and friends another £5,000 in annual contributions, and that if they do this, and the fact was made known to the present governors of the hospital, with a request that each of them would increase their own subscription by one-third and either send in the name of, or, better still, use their influence with a friend or friends to contribute annually a sum equal to another third of their present annual subscription, that the remaining £10,000 a year would be subscribed with alacrity; because every governor who takes an interest in the hospital would feel it to be a privilege to take part in a movement thus carefully thought out, which by its success would solve the financial problem of the largest of the metropolitan hospitals for many years to come, and probably for all time.

We have only ventured to indicate the outlines of the scheme by which the desired end may be most readily accomplished. There are several other minor sources through which annual income might be raised. In any case, if the committee can see their way to lead a purposeful, definite, strong effort to finance the London Hospital once and for all by undertaking to raise amongst themselves and their friends £5,000 a year, we have not the slightest doubt that the remaining £25,000 will be secured within six months from the present time. A great scheme to be successful needs the goodwill and co-operation by personal service of everybody interested in its successful accomplishment. The London Hospital has a great history; it is doing a magnificent work in the most efficient manner, and there is no man or woman of any intellectual power who can deny themselves the privilege of taking some hand in providing adequately through its agency for the needs of the sick and suffering poor of the East-end of London.

